# Transformation to Plasmablastic Lymphoma Following CD19‐Directed CAR T Cell Therapy for Refractory Diffuse Large B Cell Lymphoma: A Case Report

**DOI:** 10.1155/crip/6303492

**Published:** 2026-10-14

**Authors:** Sinan G. Khudhur, Ishaq A. Asghar, Yousif M. Abdelmoneim, Liuyan Jiang, Yao-Shan Fan, Aziza Nassar

**Affiliations:** ^1^ Department of Pathology Mayo Clinic Jacksonville Florida USA mayoclinic.org

**Keywords:** CAR T cell therapy, CD19 antigen escape, diffuse large B cell lymphoma, EBV negative, plasmablastic lymphoma

## Abstract

Plasmablastic lymphoma is a rare, aggressive large B cell lymphoma characterized by plasmablastic morphology, terminal B cell (plasma cell) differentiation, frequent loss of conventional B cell markers, and poor clinical outcomes. Although classically associated with immunodeficiency and Epstein–Barr virus (EBV) infection, plasmablastic lymphoma has also been described as an uncommon mechanism of relapse after CD19‐directed chimeric antigen receptor T cell (CAR‐T) therapy. We report a 69‐year‐old man with a remote history of chronic lymphocytic leukemia/small lymphocytic lymphoma and subsequent refractory diffuse large B cell lymphoma (DLBCL) who developed rapidly progressive, CD19‐negative plasmablastic lymphoma approximately 1 month after CD19‐directed CAR‐T therapy. Although DLBCL was of a nongerminal‐center phenotype with expression of pan‐B markers including CD19 and lack of CD138, the post‐CAR‐T transformed lymphoma demonstrated acquisition of plasmablastic morphology and CD138 expression, loss of pan‐B markers and negative for EBV by in situ hybridization and HHV8 by immunostaining. Fluorescence in situ hybridization (FISH) on the plasmablastic lymphoma showed a *MYC::IGH* fusion and low‐level MYC copy gain that were absent in the antecedent DLBCL. Disease dissemination was later confirmed on left pleural fluid cytology a few days before the patient passed away. This case highlights EBV and HHV8 negative plasmablastic transformation as a rare but important post‐CAR‐T relapse pattern and underscores the need for repeat biopsy, comprehensive paired immunophenotyping, and molecular studies in patients with atypical progression after cellular therapy.

## 1. Introduction

Chronic lymphocytic leukemia/small lymphocytic lymphoma (CLL/SLL) is the most common adult leukemia in Western countries and usually follows an indolent clinical course. A clinically significant complication is histologic transformation to an aggressive lymphoma, most often diffuse large B cell lymphoma (DLBCL), a process known as Richter transformation [1, 2]. Less commonly, transformed disease may show alternative aggressive morphologies, including plasmablastic lymphoma [3].

Plasmablastic lymphoma is an aggressive lymphoma with the morphologic and immunophenotypic features of terminal B cell differentiation. It typically shows expression of plasma cell–associated markers such as CD138 and MUM1, high proliferative activity, and reduced or absent expression of conventional B cell markers, including CD20 and PAX5. Plasmablastic lymphoma is classically associated with immunodeficiency, particularly human immunodeficiency virus infection and posttransplant immunosuppression, and many cases are EBV positive [4, 5]. However, it can also arise in immunocompetent patients and in the setting of a prior lymphoid malignancy.

CD19‐directed CAR‐T therapy has transformed the treatment landscape for relapsed or refractory B cell lymphomas. Nevertheless, relapse after therapy remains a major clinical challenge. Mechanisms of relapse include CD19 antigen loss or mutation, antigen downregulation, epitope masking, clonal selection, and lineage plasticity. Plasmablastic transformation after CD19‐directed therapy is rare but clinically important because the resulting neoplasm may be invisible to CD19‐targeted immune pressure and may require a fundamentally different therapeutic strategy. We report a case of rapidly emerging plasmablastic lymphoma following CD19‐directed CAR‐T therapy for refractory DLBCL.

## 2. Case Report

A 69‐year‐old man had a remote history of self‐reported CLL/SLL treated with 5 cycles of chlorambucil completed in 2007 and 6 cycles of bendamustine completed in 2013, followed by an approximately 12‐year remission. His medical history also included benign prostatic hyperplasia, localized prostate adenocarcinoma treated with antiandrogen therapy, intracranial hemorrhage, paroxysmal atrial fibrillation, aortic dissection, hypertension, and hyperlipidemia.

In 2024, he developed generalized weakness and loss of appetite. Imaging studies showed extensive lymphadenopathy. Biopsies of the left inguinal and subsequently left supraclavicular lymph nodes demonstrated the diffusely proliferating neoplastic lymphocytes, which expressed the pan‐B cell markers including CD20, PAX5, CD19, and CD79a; and were negative for CD138, CD10, BCL6, CD5, and cyclin D1. The neoplastic lymphocytes expressed MUM1, BCL2, and MYC, with a proliferation index by Ki‐67 of approximately 70% (Figure 1). FISH study on DLBCL was negative for *MYC*, *BCL2*, or *BCL6* gene rearrangement. There was no evidence of CLL/SLL in the background by both flow cytometry analysis and immunohistochemistry studies [6, 7]. FISH for CLL panel was completely negative. We further performed immunostaining of CD279 (PD‐1) and it was completely negative, a finding that favored de novo DLBCL rather than clonally related Richter transformation in the available clinical context [8].

**Figure 1:**
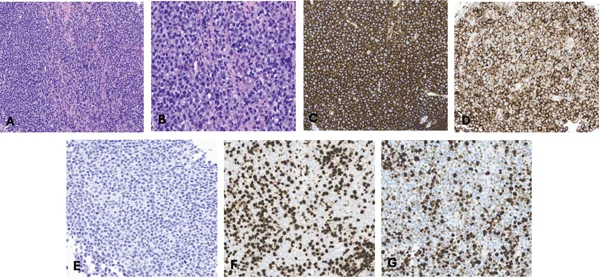
The core biopsy from left supraclavicular lymph node shows diffusely proliferating neoplastic lymphocytes with large nuclei, open chromatin, prominent nucleoli, and frequent mitosis ((A,B) H&E ×20 and ×40). The neoplastic lymphocytes are positive for CD20 and CD19 and negative for CD138 ((C–E) ×40). The neoplastic lymphocytes also showed MYC expression (70%) ((F) ×40). The overall proliferative rate by Ki‐67 is 70% ((G) ×40).

Both flow cytometry analysis and immunostaining confirmed CD19 expression on the lymphoma prior to CAR T cell therapy. After 3 cycles of chemotherapy (R‐CHOP regimen), positron emission tomography/computed tomography (PET/CT) and repeat tissue biopsy confirmed persistent disease. Because of chemotherapy‐refractory DLBCL, CD19‐directed CAR‐T therapy was administered. Approximately 1 month after therapy, follow‐up imaging demonstrated increasingly confluent, fluorodeoxyglucose‐avid soft tissue in the bilateral retroperitoneum (greater on the left), along with increased size and metabolic activity of thoracic lymph nodes, a right apical pulmonary nodule, and a right paraspinal soft tissue nodule. The overall metabolic response was consistent with progressive disease (Deauville Score 5).

A core needle biopsy (with touch preparation) of the retroperitoneal soft tissue mass demonstrated a diffuse proliferation of mononucleated cells with plasmablastic morphology and a markedly different immunophenotype from the antecedent DLBCL. The neoplastic cells were negative for pan‐B cell markers including CD20, CD19, and PAX5 but strongly and diffusely positive for CD138 and MYC with unequivocal kappa light‐chain restriction and a high proliferative rate by Ki‐67 (90%) (Figure 2). EBER in situ hybridization for EBV and immunostaining for HHV8 studies were negative.

**Figure 2:**
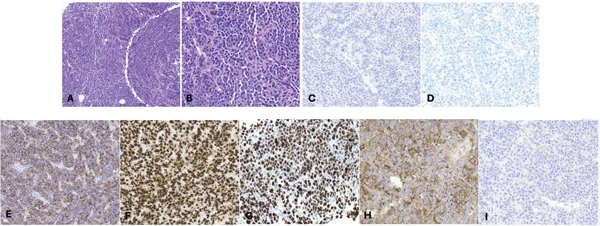
The core needle biopsy from retroperitoneal mass showed a diffusely proliferation of large neoplastic mononucleate cells with abundant cytoplasm and eccentrically located nuclei; some of them show plasmacytoid features ((A,B) H&E ×20 and ×40). Immunostains showed that they are negative for CD20, CD19; and positive for CD138 and MYC (90%) ((C–F) ×40). The proliferative rate by ki‐67 is high (90%) ((G) ×40). Kappa and lambda light chain stains demonstrated kappa restriction ((H,I) ×40).

Interestingly, the plasmablastic lymphoma demonstrated a *MYC::IGH* fusion in 53% of cells and 3–4 copies of *MYC* gene in 68% of cells, a finding not present in the antecedent DLBCL. No *BCL2* or *BCL6* rearrangements were identified. These findings suggest acquisition of *MYC*‐related genetic alterations during transformation. Molecular study by PCR for B cell gene rearrangement performed on formalin‐fixed paraffin embedded blocks of both DLBCL and plasmablastic lymphoma demonstrated identical clonal peaks, supporting clonal continuity between the two tumors (Figure 3).

**Figure 3:**
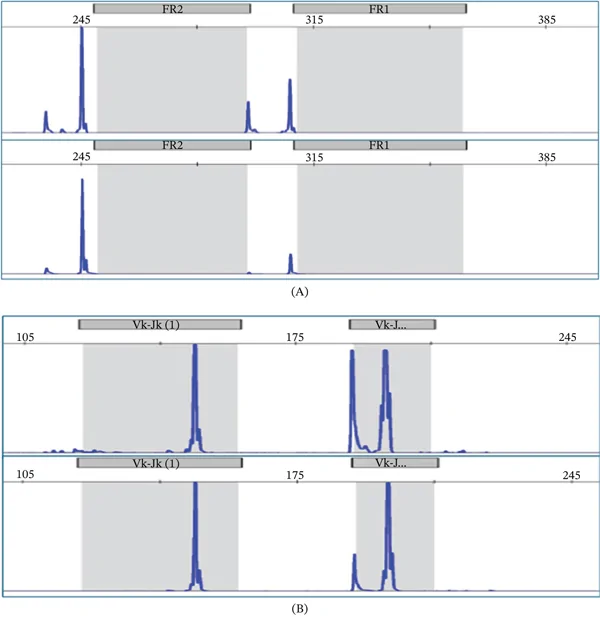
Molecular study by PCR was performed on both specimens (DLBCL and plasmablastic lymphoma [PBL]). Electropherograms demonstrate identical clonal peaks using primers targeting the immunoglobulin heavy chain (IGH) framework regions FR1 (Framework Region 1) and FR2 (Framework Region 2), as well as immunoglobulin light chain Vk‐Jk regions. The identical clonal peaks in (A) DLBCL and (B) PBL support clonal continuity between the two lymphomas.

Given the plasmablastic phenotype and the loss of B cell markers after CD19‐directed therapy, additional testing for BCMA was performed and showed strong, diffuse expression in more than 95% of neoplastic cells. Salvage therapy with teclistamab, a BCMA‐directed bispecific antibody, was initiated on the basis of the strong BCMA expression.

Approximately 1 month after the retroperitoneal diagnosis, disease dissemination was confirmed on left pleural fluid cytology (cytospin/cell block), which was positive for metastatic plasmablastic lymphoma. The patient developed worsening renal function and progressive clinical decline and elected to proceed with palliative care. He passed away approximately 1 month after the initial plasmablastic lymphoma diagnosis.

## 3. Discussion

This case illustrates a rare and clinically aggressive evolution from refractory DLBCL to plasmablastic lymphoma shortly after CD19‐directed CAR T cell therapy. The posttreatment biopsy demonstrated the key diagnostic features of plasmablastic lymphoma: a plasmablastic phenotype, diffuse plasma cell marker expression, high proliferative activity, light‐chain restriction, loss of conventional B cell markers, and absence of ALK expression [4, 5]. Notably, the lymphoma was negative for EBV and HHV8, emphasizing that the plasmablastic lymphoma may occur outside the classic viral‐associated settings. In EBV‐negative cases, distinction from plasmablastic transformation of a plasma cell neoplasm can be challenging because the two entities share substantial morphologic and immunophenotypic overlap. The diagnosis therefore depends on integration of clinical, morphologic, and laboratory findings. Features favoring a plasma cell neoplasm include a history of plasma cell myeloma, CRAB manifestations, and bone marrow involvement by clonal plasma cells. In the present case, the antecedent B cell lymphoma, absence of a serum monoclonal paraprotein, lack of marrow involvement, absence of clinicoradiological features of myeloma supported the diagnosis of plasmablastic lymphoma.

The distinguishing feature of this case is the availability of paired immunophenotypic data before and after CAR T cell therapy. The antecedent DLBCL expressed CD19 and CD20 and lacked CD138 expression, consistent with mature B cell differentiation. In contrast, the post‐CAR‐T cell plasmablastic lymphoma lost CD19, CD20, PAX5, and CD79a while acquiring diffuse CD138 expression and plasmablastic morphology; yet PCR study proved the clonal continuity of the two neoplasms. This striking immunophenotypic shift under CD19‐directed therapeutic pressure provides direct evidence of lineage plasticity and represents a rare mechanism of post‐CAR‐T cell relapse.

Relapse after CD19‐directed CAR T cell therapy may be mediated by several mechanisms, including CD19 mutation, alternative splicing, epitope masking, antigen downregulation, or selection of antigen‐negative subclones [9–12]. Lineage shift represents a particularly challenging form of immune escape because the tumor may lose the lineage‐defining antigen targeted by therapy while acquiring a different differentiation program. The immunophenotypic shift in this case—loss of CD19 and other B cell markers with acquisition of plasma cell differentiation markers—is consistent with this concept and provides a plausible explanation for resistance to CD19‐directed therapy. True antigen loss and epitope masking cannot be fully distinguished by immunohistochemistry alone, and both remain possible.

A further molecular correlate of transformation was the acquisition of *MYC* abnormalities. The plasmablastic lymphoma demonstrated *MYC::IGH* fusion with *MYC* copy gain, whereas the antecedent DLBCL was negative for these changes. These findings suggest acquisition of MYC‐related genetic alterations during disease evolution and may have contributed to the aggressive clinical behavior of the transformed lymphoma.

Whether the plasmablastic lymphoma represents clonal evolution of the preceding DLBCL, transformation from an underlying CLL/SLL clone, or emergence of a therapy‐selected secondary clone is best addressed by comparative molecular studies. The identical IGH clonal peaks from PCR studies support clonal continuity between the DLBCL and the plasmablastic lymphoma. However, comprehensive paired next‐generation sequencing and copy‐number profiling—which would more definitively establish clonal relatedness and delineate the molecular pathways associated with antigen loss and plasmablastic differentiation—were not able to perform because the tissues from both core needle biopsies were not sufficient for adequate DNA extraction [9–12].

A particularly important finding in this case was the strong and diffuse expression of BCMA in the plasmablastic lymphoma. BCMA is a member of the tumor necrosis factor receptor superfamily that is highly expressed in plasma cells and plasma cell neoplasms and is an established therapeutic target in multiple myeloma. Emerging reports suggest that BCMA may also be expressed in a subset of plasmablastic lymphomas, creating a potential therapeutic opportunity in an otherwise treatment‐refractory disease [13, 14]. The initiation of teclistamab in this patient was biologically rational given the strong BCMA expression, although the patient′s progressive clinical decline and death approximately 1 month after diagnosis precluded any meaningful assessment of response.

This case has several practical implications for surgical pathologists and hematopathologists. First, atypical or rapidly progressive disease after CAR T cell therapy should prompt repeat tissue sampling rather than assuming conventional relapse. Second, post‐CAR‐T biopsy workup should include a broad immunophenotypic panel that evaluates retained or lost B cell lineage markers (including the CD19 target), plasma cell differentiation, EBV and HHV8 status, light‐chain restriction, proliferation index, and targetable antigens such as BCMA when plasmablastic differentiation is present. Third, molecular comparison between pre‐ and post‐CAR‐T specimens should be pursued whenever tissue is available and the patient′s condition permits, because clonal relatedness and acquired alterations such as MYC rearrangement may carry prognostic and therapeutic implications. Finally, the case supports further study of BCMA‐directed therapies, including bispecific antibodies and BCMA‐directed CAR T cells, in BCMA‐positive plasmablastic lymphoma [13–15].

## 4. Conclusion

Plasmablastic lymphoma is a rare but important pattern of relapse or transformation after CD19‐directed CAR T cell therapy. The present case demonstrates rapid emergence of a CD19‐negative, BCMA‐positive plasmablastic lymphoma—with documented loss of CD19 relative to the CD19‐positive antecedent DLBCL and newly acquired MYC abnormalities—after treatment for refractory DLBCL. Recognition of this phenomenon requires repeat biopsy and comprehensive paired immunophenotyping, particularly when clinical progression is atypical for conventional relapse. Strong BCMA expression may identify a therapeutic vulnerability, but prospective studies and molecularly annotated case series are needed to define the biology, clonal relationships, and optimal treatment of post‐CAR‐T plasmablastic transformation.

## Funding

No funding was received for this manuscript.

## Conflicts of Interest

The authors declare no conflicts of interest.

## Data Availability

Data sharing is not applicable to this article as no datasets were generated or analyzed during the current study.
